# Further Remarks on Phthisis Pulmonalis

**Published:** 1817-08

**Authors:** Isaac Buxton


*For the London Medical and Physical Journal.*

### *Further Remarks on Phthisis Pulmonalis;* by Isaac Buxton, M.D.

IN Dr. Sutton's reply to my letter, published in your Number for June, p. 456, are some remarks which relate to me individually. As the personal dispute of two combatants can be very little interesting to the public at large, I shall endeavour to reply to that part of his letter as briefly as possible.

Dr. S. informs me, that he has no personal enmity towards myself. Judging of his feelings towards me by my own towards him, I never imagined that any such motive influenced either his heart or his pen.

He conceives that I am displeased with him for calling my opinions in question. In this I beg to assure him that he is mistaken. A writer who publishes his opinions is like a man who gives a general challenge; and any one, or every one, has the privilege of entering the lists with him.

Dr. S. asserts his full liberty of "communicating any thing further on pulmonary consumption that would interfere with my opinions." Without doubt, he, or any other individual, is perfectly at liberty to do this. But it did appear to me, and still does appear, rather extraordinary, that, in his communication in March 1816, p. 182, he should express a wish that the discussion between us might cease, and, either in the same paper, or in a paper published some months afterwards, should bring forward fresh matter, which was professedly intended to bear on the questions at issue between us, and to diminish the force of the evidence which I had produced. This must demand a reply; and, consequently, the wish and the procedure appear inconsistent with each other.

In your Journal for May last, p. 362, in replying to Dr. Sutton's observations respecting Sicily and Malta, I mentioned I had before shewn, that, in quoting Irvine on the Diseases of Sicily, Dr. Sutton's "supposed proof depends entirely on his separating particular passages; and, by detaching them from their situation, giving them a force, which, in their connexion, they do not possess." To this Dr. S. answers, p. 462, "I really believe he (Dr. Buxton) thinks so; but, if the reader of this note can confide in my assertion, I will assure him, that I have not employed myself in any such inferior work. I have laid always more stress upon absolute facts in the writings I have alluded to, than on

 opinion, opinion, or on opinions inferred from fact. I have, therefore, treated opinions with less importance than facts; and have paid little deference to one part of an author's book, and placed great stress on another." Were this merely a question whether my assertion or Dr. Sutton's was to be credited, the discussion would be of little consequence to the profession; but, as it involves a medical fact, it may not be altogether unworthy of attention. Dr. S. has stated the case in your last Number, and has given a reply, as if I had brought this forward as a general charge, whereas my accusation, in this instance, relates merely to Dr. Irvine's book on the Diseases of Sicily. He likewise omits to observe, that this is a deduction from proofs which I had formerly given, and which the reader may find in your Journal for Nov. 1815, p. 371, 2. If Dr. Sutton were to answer the arguments there brought forward in proof of my assertion, it would, perhaps, produce a stronger effect than simply denying the charge. A refutation, however, he has not attempted. On this occasion, Dr. Sutton appears to have forgotten that Dr. Irvine gives no positive numbers or proportions, but uses the word *frequent*, and others of a similar nature. Now the expressions *frequent* and *unfrequent*, *common* and *uncommon*, are relative terms, and are only to be judged of by the context and by other circumstances; so that, in a case of this kind, it is impossible, from detached unconnected passages, to draw a fair conclusion; but the general tenor of the book must necessarily be consulted. It is by overlooking this observation that Dr. Sutton has drawn unfair conclusions.

The commencement and the end of the above quotation from Dr. Sutton appear to me inconsistent with each other. The first denies, and the latter acknowledges, the truth of my charge. I had said, in reference to Dr. Irvine's book, that Dr. S., by "separating particular passages," &c. Dr. Sutton at first denies this; but afterwards allows, that he has "paid little deference to one part of an author's book, and placed great stress on another." Now the charge which I have made, though conveyed in other words, is precisely this, that Dr. Sutton has "paid little deference to one part" of Dr. Irvine's book, and "has placed great stress on another;" and thus has opposed the general tenour of that work.

In the above quotation, Dr. Sutton appears to maintain, that no attention should be paid to inferences or opinions. Unsupported inferences are certainly unworthy of attention. Few persons will reject the opinions of others respecting facts, unless those opinions are different from their own, provided the reporter is a man whose veracity has not been impeached, impeached, provided his opportunities of observation are sufficient, and provided he has no particular theory to support. For the last reason, neither Dr. Sutton's opinions nor mine are to be received implicitly. Yet, though Dr. Sutton has this dislike to inference, he can, on some occasions, have recourse to inference, and that with very insufficient premises. Of this I gave an example in my last communication, where I shewed that, on the supposed testimony of one writer respecting Sicily, Dr. Sutton drew an inference as to Malta, which inference was in direct opposition to the testimony of a writer respecting Malta itself. And I shall immediately have occasion to point out another inference, drawn by Dr. Sutton from insufficient premises.

In your Journal for last February, p. 96, Dr. Sutton inferred the general frequency of consumption in Malta, because the returns of one year shewed, that one death in five among our troops in that island was occasioned by this disease. To this I replied, p. 363, that the army consisted of persons between the ages of ten and forty: that this, consequently, by excluding all infantile and senile diseases, could not be taken as a fair general standard; and that, by registers of deaths in civil life, kept according to the ages, it was shewn, that double the proportion died of consumption between ten and forty, of those who died of this disease when all ages were included. To this Dr. Sutton has rejoined, p. 461, "None of infirm health, or consumptive, are admitted to be soldiers; and it is to be observed, that a more than equal share of those who die of consumption are of the class of those who cannot be admitted into the army. Next it may be observed, that women are, upon the whole, more liable to consumption than men. These two causes appear to effect an equality between the whole of the deaths in civil life, compared with those by consumption, and such as happen among the military in this country, in the total, compared with the same." In other words, if I understand the meaning of this passage, that the proportion of deaths by consumption among the military is only half what it is among males of the same age in civil life, or equal to the general proportion in civil life when all ages are included. This is the inference. Let us examine if the premises are equal to the support of such an inference. That more women are affected with consumption than men, rests on Dr. Sutton's assertion without proof; and therefore must be considered as an unsupported assumption. But, allowing it to be correct, the qualifying terms which Dr. Sutton employs, "*on the whole*," appear to imply that the difference is merely trifling even in his estimation.

With

With respect to the military, the same remark may be made, that there is assertion without proof being offered by Dr. S. That no person whose health is actually much deranged, or who is positively in a consumption at the time of presenting himself, is admitted into the army, I can easily credit. But, if an inspector of recruits were ever so desirous of excluding from the army all who had a tendency to consumption, so difficult would be the task, that, in many instances, he must be deceived. Also, during war, I have myself known, and have been informed on good authority, that men are passed, on account of the difficulty of procuring recruits, who, during peace, would be rejected. That in time of war men are admitted into the army of a decidedly consumptive disposition, I know by experience; and have been assured, that such is the fact, by gentlemen whose opportunities of gaining this kind of knowledge are much greater than I possess. I have also been informed by an inspector of recruits, that, during war, he was not required to be, nor was he actually, particular in this point; and that, in fact, he passed all, with scarcely any exception, who were free from disease at the time of examination. As this is diametrically opposite to Dr. Sutton's unproved assumption, his inference must fall to the ground; and, unless Dr. S. can give stronger proof than that contained in the above quotation, we must not allow that the deaths by consumption in the army are only half those which occur among males of the same age in civil life.

But to strengthen this supposition, Dr. Sutton says, p. 461, that Sir James M'Grigor "asserts that deaths by consumption among the troops in Great Britain amount to one in five of the deaths by other diseases." And a little lower, in the same page, he refers to this as one of the "decided matter-of-fact statements of Sir James M'Grigor." But, unfortunately, this is not the matter-of-fact statement of Sir James M'Grigor, but the statement of Dr. Sutton. In the Transactions of the Medico-Chirurgical Society, vol. vi. p. 440, Sir James M'Grigor says, "In England this disease (consumption) is as frequent and fatal in the army as it is in civil life: in Spain and Portugal we found it of much more rare occurrence;" and, speaking "of the army in England, under ordinary circumstances, and when no epidemic or contagious disease prevailed, I found the deaths from consumption to amount to one-fifth, one-fourth, and, in some regiments, even as high as one half, of the whole mortality. I have given these quotations, that each may speak for itself. Dr. Sutton asserts, that the deaths by consumption among the military in England are one in five. Sir James M'Grigor evidently, in in the first part of the quotation from his paper, had not the intention of accurately stating the exact proportion; but says, in general terms, that consumption is "as frequent and as fatal in the army as it is in civil life," without particularly determining whether he meant among persons of the same age, or of all ages indiscriminately, in civil life. And I have before shewn, that this circumstance occasions a very material difference. Sir James subsequently lays a statement before the reader, which gives the smallest proportion of deaths by consumption at one fifth, and the highest at one half. Dr. Sutton's assumption, therefore, founded on the supposed authority of Sir James M'Grigor, that the smallest of these proportions, viz. one fifth, was the universal proportion, is plainly an error. I might with nearly as much propriety assume, on the same authority, that one half was the general proportion in the army: whereas, it is evident that the average must be somewhere between these two extremes.

In reckoning the proportion of soldiers who die of consumption in England, it should be recollected that some affected with this complaint are discharged on account of their disease, and, consequently, that their deaths are not enumerated in the returns of their respective regiments. What effect this circumstance would have in altering the proportion of the deaths by this disorder among the military, I am not able to say; but, that it would affect the proportion in a greater or smaller degree, I am certain, having repeatedly witnessed the fact to which I allude. Three men were recently under my care for consumption, in the Infirmary for Diseases of the Lungs, all of whom had been discharged from the army on account of their disorder. Two of these men had fought at the battle of Waterloo, of whom one was of a decidedly consumptive make, being fair, thin, and narrow-chested.

In his last communication, Dr. Sutton appears desirous of making the military returns the exclusive foundation of his conclusions respecting the diseases of every country and climate; and those returns only from a very small number of stations. Documents of this description are, undoubtedly, of high importance; but I see no reason for limiting our observation to this class of persons alone, more especially when so many able writers on the diseases of different countries exist, whose observations are not confined to any particular cast. Indeed, the taking any one set of persons exclusively, and arguing from them alone to the general body of men, women, and children, inhabiting any country, must necessarily lead to false results. I need not enlarge on this topic, as it cannot fail of being obvious to every medical

2 man, man, that English soldiers are too exclusive a body of men, and too peculiarly circumstanced, to enable us from them alone to speak with any degree of accuracy respecting the diseases and deaths occurring among the inhabitants in general of a foreign country, in which these men happen to be stationed.

I believe I have now noticed all the points of Dr. Sutton's last letter which bear on the controversy between us. The remainder, not relating immediately to this subject, I have not thought it necessary to answer.

*New Broad-street;*

*June* 24, 1817.

---

We feel greatly indebted to our learned and ingenious correspondents. An enquiry, conducted by men of such experience, cannot but elicit important information; but the number of candidates for notice obliges us to request that the controversy may now cease, and that all future communications on this subject may be confined to statistical facts.—EDIT.

---

*For the London Medical and Physical Journal.*

